# The Hospital World

**Published:** 1910-09-17

**Authors:** 


### THE HOSPITAL WORLD.

### Elections and Appointments.

Mrs. Heywood Thompson has qualified as a life governor of the Cumberland Infirmary. Mr. Beaty has been elected to the Committee of Election.

Colonel the Hon. H. W. Forbes Trefusis has been elected president of the Royal Cornwall Infirmary for the ensuing year, and the Rev. W. E. Fenwick, Mr. G. T. Petherick, Mr. A. C. Polwhele, Mr. G. Cookson, and Mr. E. Heard have been added to the committee of management.

Mr. Frank Brown, J.P., has been elected president of the Stockton and Thornaby Surgical Hospital, in succession to the late Alderman W. Anderson. Mr. Thomas Shaw, Mr. Robert Atlay, Mr. Charles Fuller, and Mr. Charles F. Philpotts have been elected life governors. Mr. Leonard Ropner, J.P., has been appointed a trustee and Mr. W. Whitwell, J.P., has been re-elected hon. treasurer, with Mr. R. Reed as his hon. assistant.

The annual election of officers at the Shirley Children's Hospital and Dispensary for Women, Shirley, Southampton, has resulted as follows: Chairman, Capt. J. Henderson, R.N.; Committee, Miss Carlyon, Mrs. Cooper, Rev. G. G. Elton, Mrs. Henderson, Mrs. Hutchinson, Miss McQuhae, Mrs. Murton, Capt. Nicholls, Mr. P. Milne Stewart, Mrs. Saunders, Mrs. Sinkins, Mrs. Spooner, Mrs. Thomsett, and Mrs. Neville Ward; hon. treasurer, Mr. A. Spooner; hon. secretary, Mr. B. Winn Ford; hon. auditor, Mr. F. Woolley.

### Personalia.

The King and Queen have given their patronage to the Evelina Hospital for Sick Children, Southwark, and to the City of London Hospital for Diseases of the Chest, Victoria Park, E.

The Guildford, Godalming, and Woking Joint Hospital Board is full of regret that its friend and former President, Dr. W. G. Niall, is leaving Guildford, and thereby severing his connection with local hospital work. In acknowledging the compliments that were addressed to him at a meeting of the board held a few days ago Dr. Niall remarked that his year of presidency, indeed, the whole period of his membership, had been marked by the most pleasant relations, which he would not forget wherever his work took him. He thanked the matron of the hospital and the officials of the board for the kindness he had received from them so constantly.

